# Design of a Low-Scattering Dual-Band Metasurface Array Antenna Using Characteristic Mode Theory

**DOI:** 10.3390/ma19173603

**Published:** 2026-08-25

**Authors:** Jing Zou, Huanhuan Yang, Tong Li, Tianhao Wu, Zixiang Pan, Can Li, Zexu Guo

**Affiliations:** Information and Navigation College, Air Force Engineering University, Xi’an 710077, China; 15570420129@163.com (J.Z.); 13103281915@163.com (T.W.); pzxmircowave@163.com (Z.P.); lc000210@163.com (C.L.); 14291004@bjtu.edu.cn (Z.G.)

**Keywords:** characteristic mode theory, metasurface array antenna, dual-band radiation, radar cross-section reduction

## Abstract

This work proposes a Characteristic Mode Theory (CMT)-guided method for the co-design of radiation and scattering performance of a low-scattering dual-band metasurface array antenna. Conventional design approaches generally treat radiation design and RCS reduction as two separate targets. In contrast, the proposed method leverages the differences in the spatial distributions of radiation and scattering characteristic modes. Dual-band radiation modes are constructed and excited within the central region of the metasurface, while the edge and corner regions are locally reconfigured to suppress dominant scattering modes without significantly perturbing the radiation-mode current distributions. In this way, dual-band radiation and broadband RCS reduction are simultaneously realized within a single metasurface aperture. A systematic radiation–scattering co-design workflow driven by characteristic-mode parameters is established. Modal significance (MS), radiation-mode current distributions, and modal radiation patterns are used to regulate the target radiation modes and determine the feeding configuration. Modal weighting coefficients (MWCs) under plane-wave illumination are used to identify the dominant scattering modes, while the corresponding scattering-mode current distributions are used to determine the structural modification regions and corresponding modification strategies. The antenna developed using the proposed method integrates dual-band radiation and broadband RCS reduction within a single low-profile configuration. Measured results demonstrate that the antenna covers two operating bands of 3.08–3.10 GHz and 3.12–3.18 GHz, with peak gains of 16.0 dBi and 16.4 dBi, respectively. Within the 6.5–10.5 GHz band, more than 10 dB monostatic RCS reduction is achieved under both x- and y-polarized plane-wave illumination.

## 1. Introduction

With the rapid advancement of wireless communication, radar detection, telemetry and navigation, as well as multifunctional RF systems, antennas are increasingly required to operate across multiple frequency bands within constrained mounting spaces [1,2,3]. Compared with deploying multiple standalone antennas, dual-band antennas can simultaneously reduce the antenna count and occupied aperture, thereby improving system integration and reducing the structural load on the carrier platform [4,5,6]. Meanwhile, when antennas are integrated into low-observable platforms, such as aircraft, unmanned aerial vehicles, and vessels, they serve not only as signal transmitting/receiving modules but also as prominent electromagnetic scattering sources [7,8,9]. Accordingly, developing antennas that integrate dual-band radiation and low-RCS performance is essential for compact, highly integrated, low-observable RF systems [10,11].

In recent years, various design strategies have been proposed, including covering dual-band antennas with polarization-rotating metasurface radomes [12], loading polarization-rotating metasurfaces [13] or phase-coding metasurfaces around radiators [14], and adopting multilayer functional metasurface configurations [15]. In these existing schemes, low-RCS structures are typically introduced after the antenna radiation performance has been established. Iterative parameter optimization is subsequently performed to mitigate the perturbation of the original radiation-mode current distributions caused by these low-RCS structures. As a result, radiation design and RCS reduction are generally treated as two separate design tasks in most existing works. Therefore, there is a need to establish a unified co-design framework for dual-band radiation and broadband RCS reduction and to further reveal the underlying physical mechanisms linking radiation and scattering characteristics.

Characteristic Mode Theory (CMT) has been extensively adopted for radiation design and scattering-mode analysis. For instance, Refs. [16,17,18,19] employ CMT to guide radiation-mode construction, modal excitation, and feeding configuration. Refs. [20,21,22] mainly employ CMT to identify dominant scattering modes and guide structural modifications for RCS reduction. In Ref. [23], a phase-gradient metasurface is first designed to achieve ultra-wideband RCS reduction; subsequently, CMT is employed to determine the feeding configuration, and the antenna structure is further optimized based on modal significance (MS) and modal weighting coefficients (MWCs) to enhance the RCS-reduction performance without significantly degrading the radiation performance.

Unlike the above studies, in which CMT is primarily applied to radiation or scattering design in separate or sequential stages, this paper proposes a CMT-guided co-design method for radiation and scattering performance. The proposed method is validated using a low-profile metasurface array antenna. The main contributions of this work are outlined as follows:

1. A CMT-based design strategy for dual-band low-scattering antennas is presented. By analyzing the spatial differences between radiation and scattering characteristic modes, targeted modal regulation is performed in the central region of the metasurface to realize dual-band radiation, while the outer-edge and corner regions are used for scattering control. In this way, dual-band radiation and scattering suppression are simultaneously realized within the same metasurface aperture.
2. A step-by-step radiation–scattering co-design workflow driven by characteristic-mode parameters is established. Modal significance, radiation-mode current distributions, and modal radiation patterns are used to regulate the target radiation modes and determine the feeding configuration. The MWCs under plane-wave illumination are used to identify the dominant scattering modes. The corresponding scattering-mode current distributions are then used to determine the regions to be modified and the associated structural modification strategies. Therefore, each step of the proposed procedure is driven by modal parameters with clear physical significance, thereby reducing the dependence on empirical trial and error, blind parameter sweeps, and global optimization.
3. The metasurface antenna designed under the guidance of CMT integrates dual-band radiation and broadband RCS reduction within a single low-profile structure. Measured results demonstrate peak gains of 16.0 dBi and 16.4 dBi for the two operating bands, respectively, and more than 10 dB monostatic RCS reduction is achieved across the target frequency band.

## 2. Antenna Design

### 2.1. Design of Antenna Radiation Characteristics

**Figure 1:**
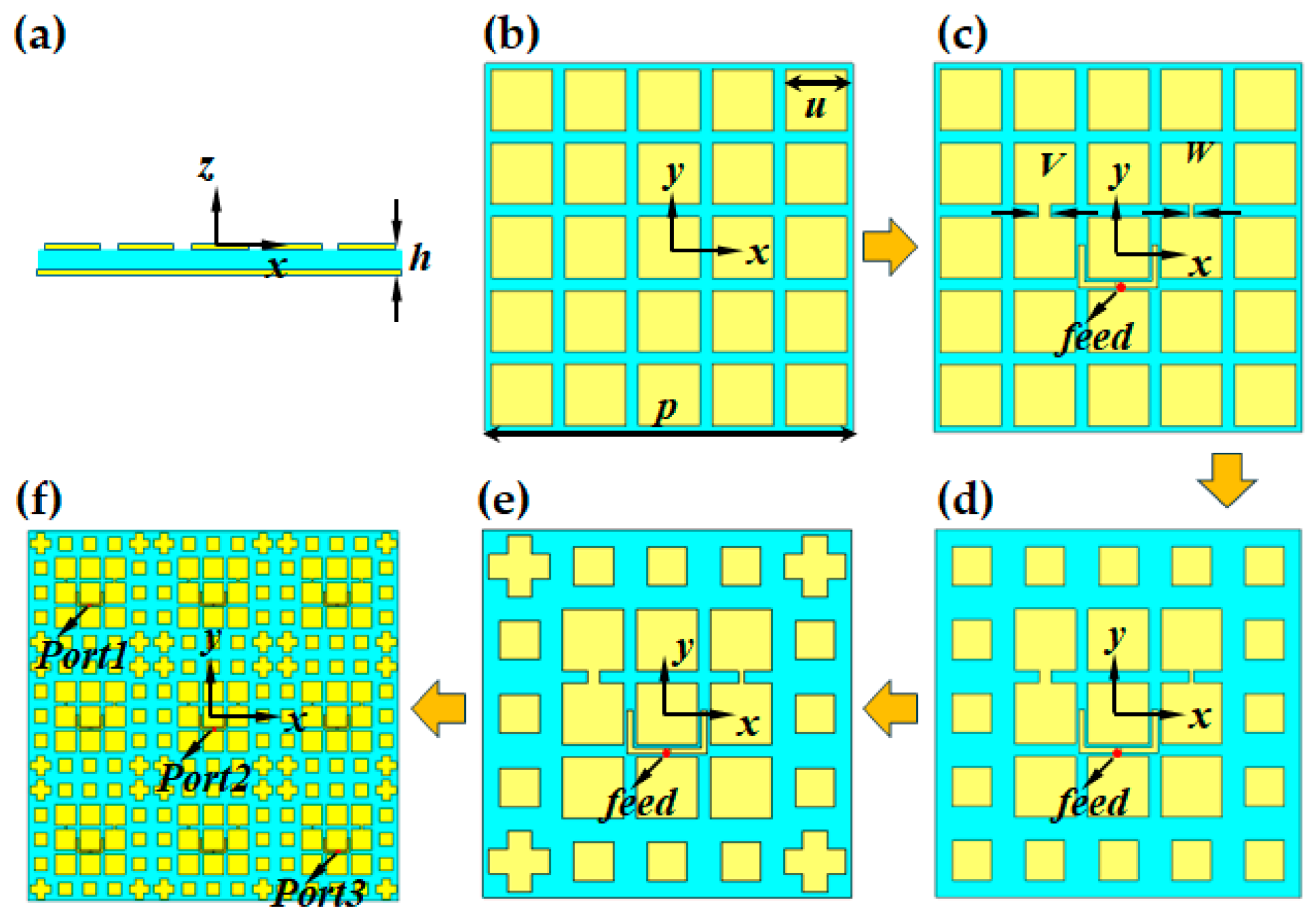
Schematic illustration of the structural evolution from the metasurface to the array antenna: (**a**) side view of the initial metasurface; (**b**) top view of the initial metasurface; (**c**) antenna element 1; (**d**) antenna element 2; (**e**) antenna element 3; and (**f**) 3 × 3 array antenna.

A uniform patch-type metasurface provides abundant modal degrees of freedom within a confined aperture. This characteristic facilitates mode selection and regulation for the co-design of radiation and scattering performance. Accordingly, a 5 × 5 uniform patch-type metasurface is selected as the design platform. The initial metasurface structure is shown in Figure 1a,b. It consists of an upper metallic patch layer, a dielectric substrate, and a bottom metallic ground plane. Each metallic patch measures 10.0 mm × 10.0 mm. The dielectric substrate has overall dimensions of 60.0 mm × 60.0 mm, a thickness of 3.0 mm, a relative permittivity of 2.65, and a loss tangent of 0.002. Unlike conventional patch-antenna design approaches that rely primarily on geometric resonances, the proposed design is based on the inherent electromagnetic modes of the structure. Characteristic mode analysis is then performed to identify radiation modes that can be effectively excited, and the desired radiation performance is achieved through appropriate excitation.

**Figure 2:**
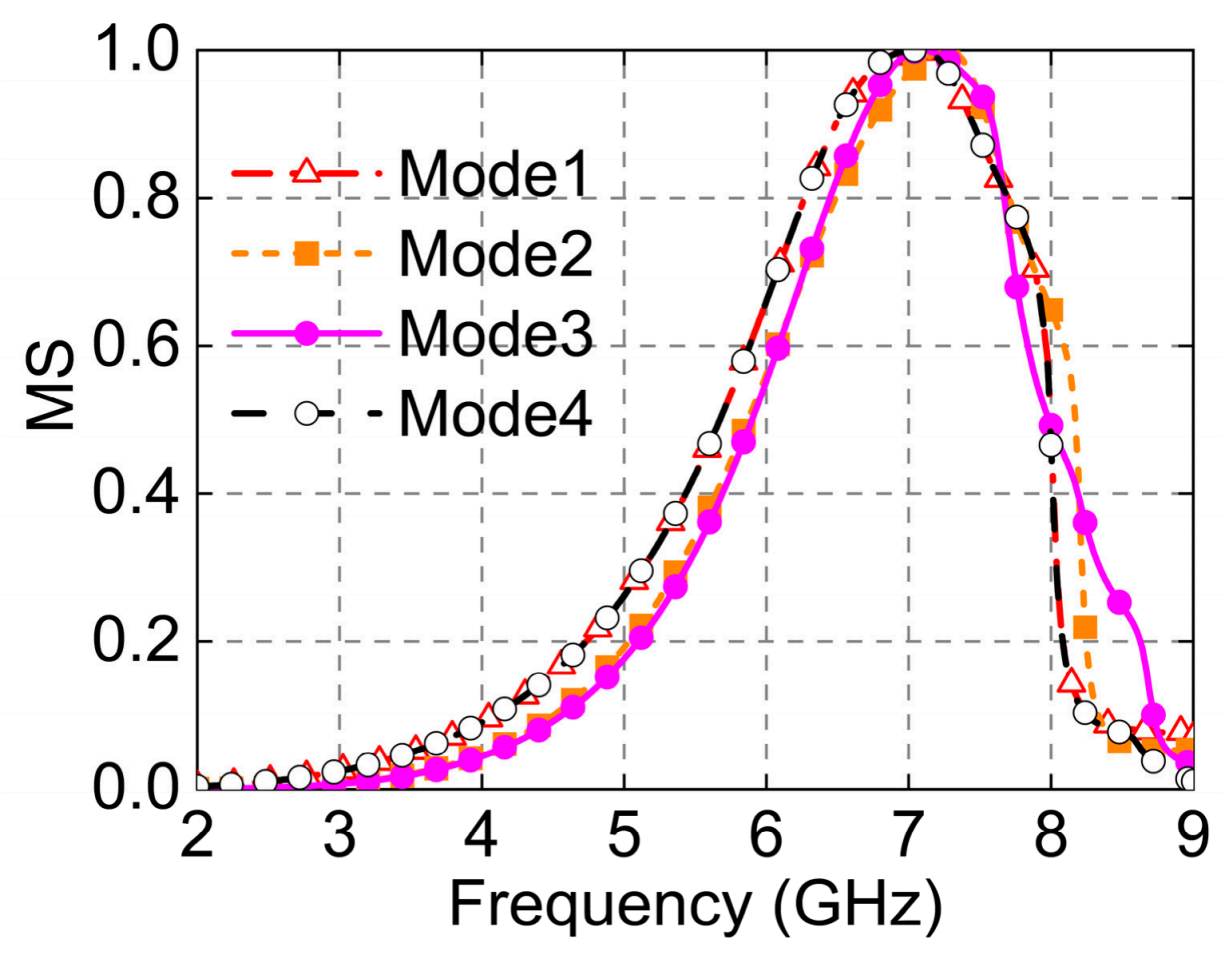
Modal significance curves of the initial metasurface.

**Figure 3:**
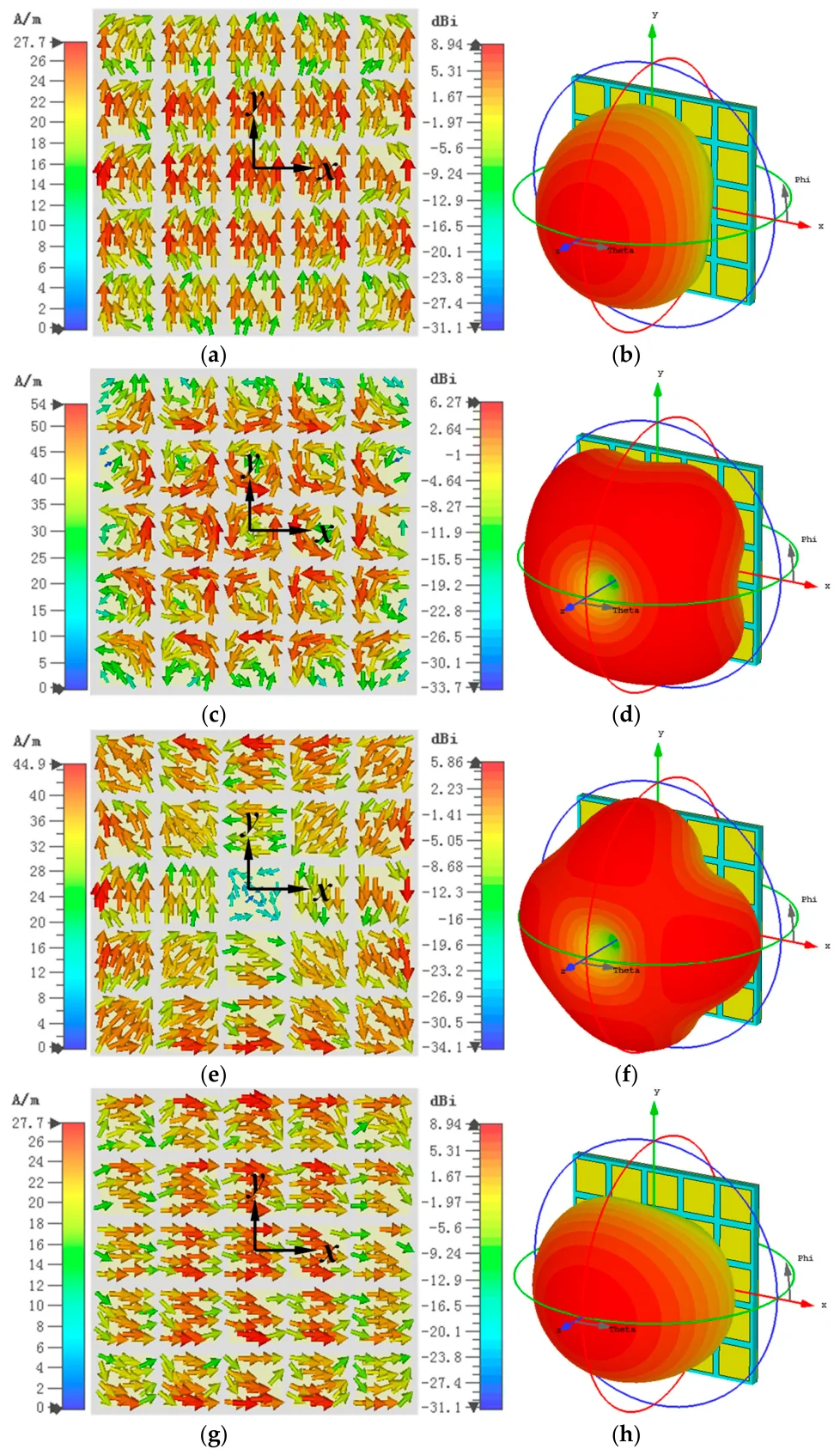
Modal current distributions and far-field radiation patterns of the initial metasurface at 7.0 GHz: (**a**,**b**) Mode 1; (**c**,**d**) Mode 2; (**e**,**f**) Mode 3; and (**g**,**h**) Mode 4.

All simulations in this work were performed using CST Studio Suite 2024, and the simulation parameters have been repeatedly validated by our research team. To characterize the inherent resonant properties of the initial metasurface, its modal significance (MS) was first calculated, as shown in Figure 2. MS quantifies the resonance level of a characteristic mode at a given frequency. An MS value approaching unity indicates that the mode is close to resonance. Around 7.0 GHz, four characteristic modes with relatively high MS values are identified. Figure 3 presents the modal current distributions and far-field radiation patterns of these modes. Mode 1 and Mode 4 form a pair of orthogonally polarized degenerate modes, with their main lobes directed along the surface-normal direction. Accordingly, these modes are well suited for broadside radiation. In contrast, Mode 2 and Mode 3 exhibit pronounced radiation nulls in the broadside direction. Consequently, Mode 1 was selected as the target radiation mode for the subsequent design, and the structural modifications were guided by its modal current distribution.

To realize dual-band radiation while retaining broadside radiation capability, local bridging patches are introduced around the high-current region of the target mode. As shown in Figure 3a, the modal current of Mode 1 flows primarily along the *y*-axis and is concentrated on the central patches of the metasurface. Based on this modal current distribution, two nearly symmetric bridging patches are introduced in the central region. The widths of the left and right bridging patches are defined as V and W, respectively, as shown in Figure 1c. These bridging patches improve the continuity of induced currents between neighboring patches. Furthermore, varying the widths of the bridging patches modifies the current paths, thereby facilitating the realization of dual-band radiation.

**Figure 4:**
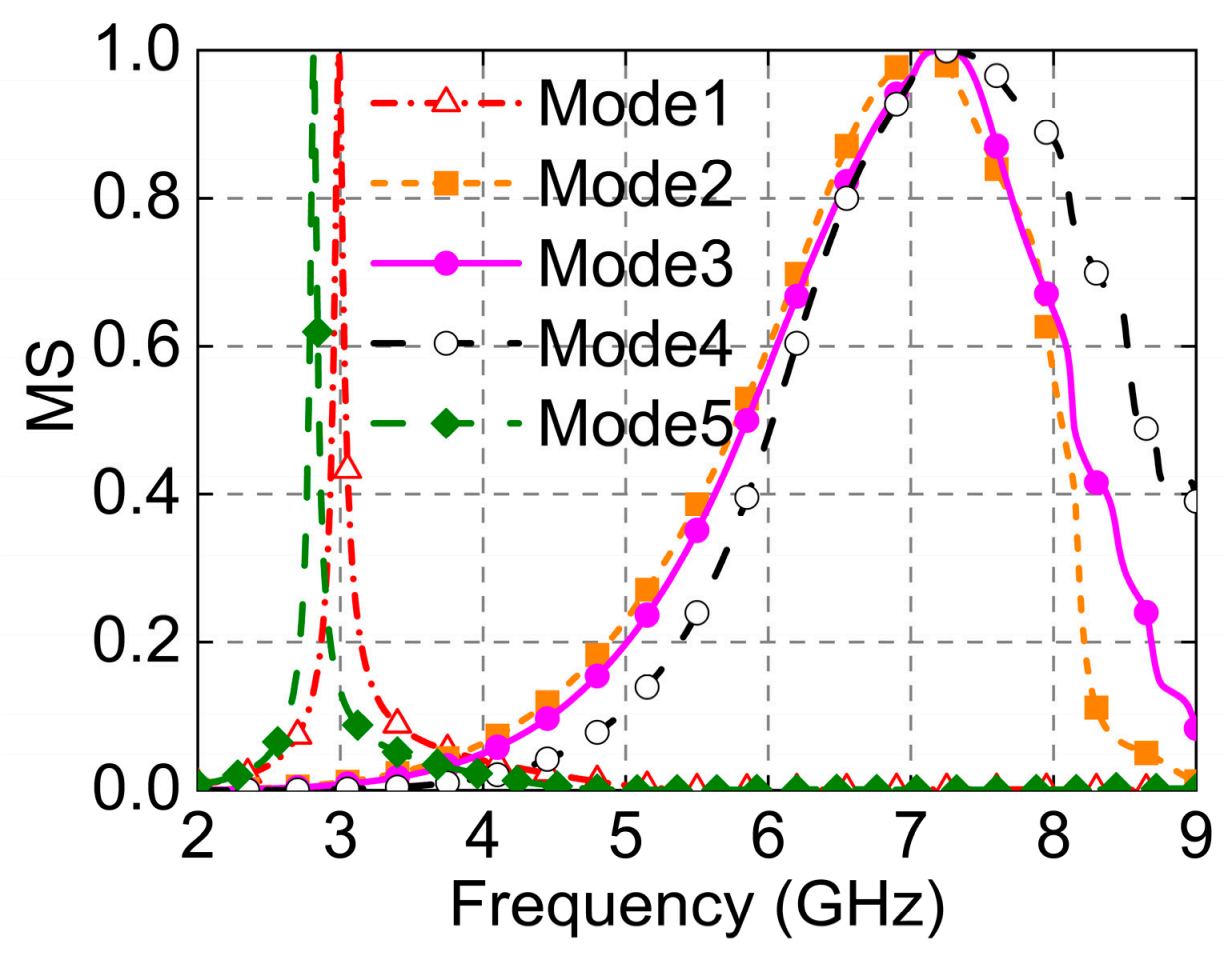
Modal significance curves of the modified metasurface.

**Figure 5:**
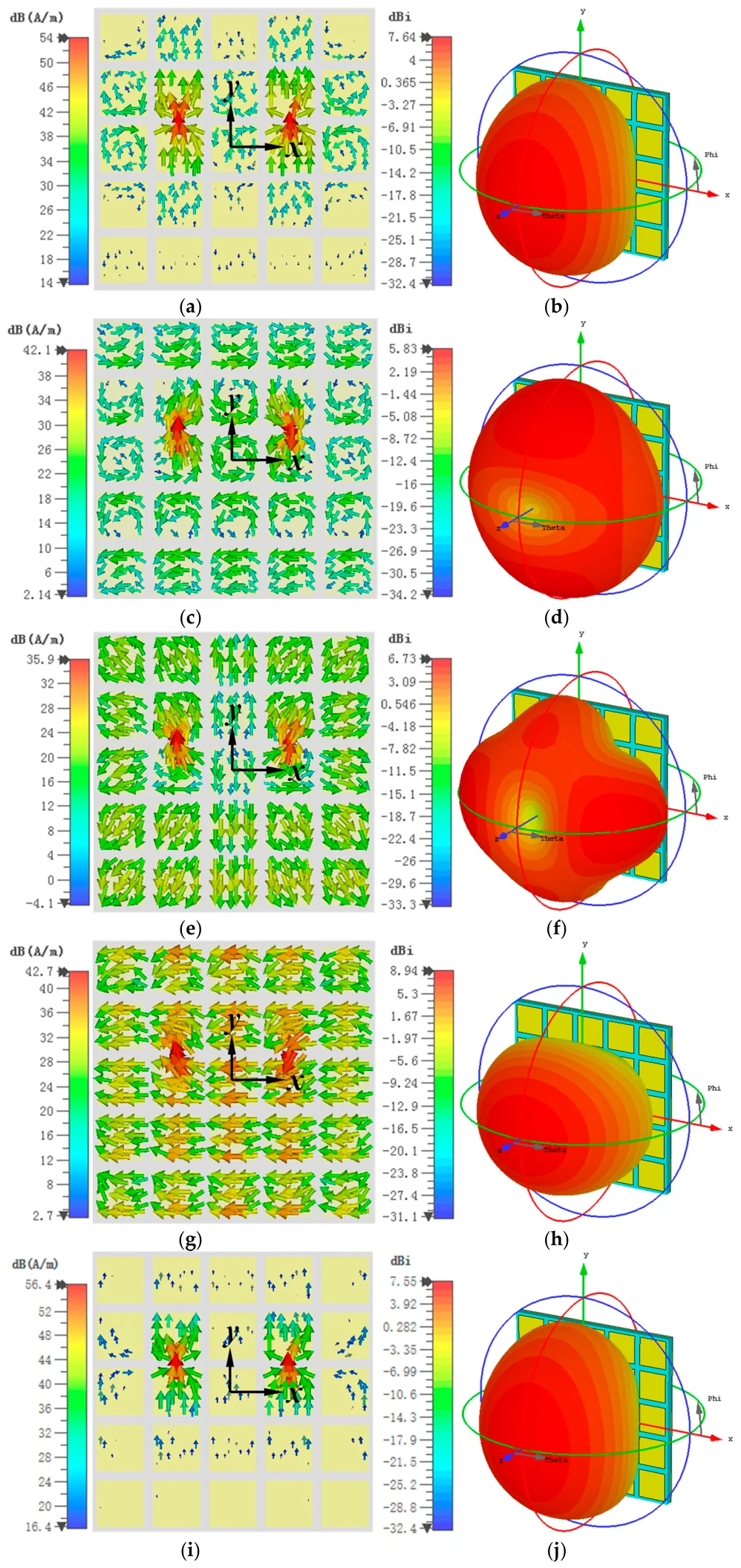
Modal current distributions and modal radiation patterns of the modified metasurface: (**a**,**b**) Mode 1 at 3.0 GHz; (**c**,**d**) Mode 2 at 7.0 GHz; (**e**,**f**) Mode 3 at 7.0 GHz; (**g**,**h**) Mode 4 at 7.0 GHz; and (**i**,**j**) Mode 5 at 2.8 GHz. In each pair, the modal current distribution and radiation pattern are shown, respectively.

Characteristic mode analysis was then performed on the modified metasurface, and the corresponding MS curves are shown in Figure 4. Compared with the initial structure, the modified metasurface exhibits two characteristic modes with relatively high MS values around 3.0 GHz, while characteristic modes similar to those of the initial structure are retained near 7.0 GHz. Figure 5 presents the modal current distributions and modal radiation patterns of the modified metasurface. Mode 1 and Mode 5 exhibit current distributions dominated by y-directed currents, together with central symmetry and broadside radiation characteristics. These results indicate that the bridging patches introduced according to the modal current distribution of the target mode preserve the fundamental current profile of the original radiation mode while effectively tuning its resonant characteristics.

**Figure 6:**
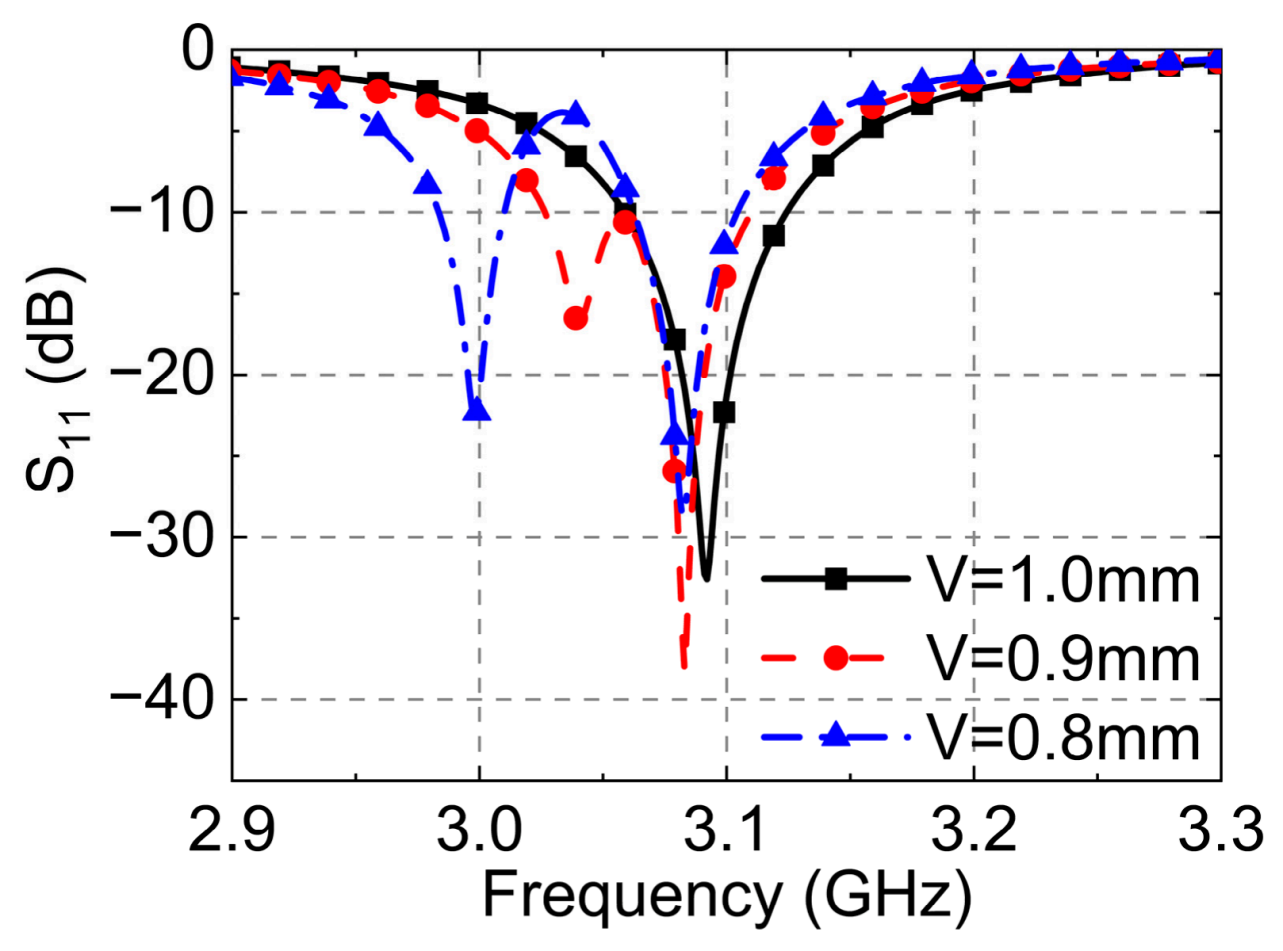
Reflection coefficients under different values of V with W = 1.0 mm.

**Figure 7:**
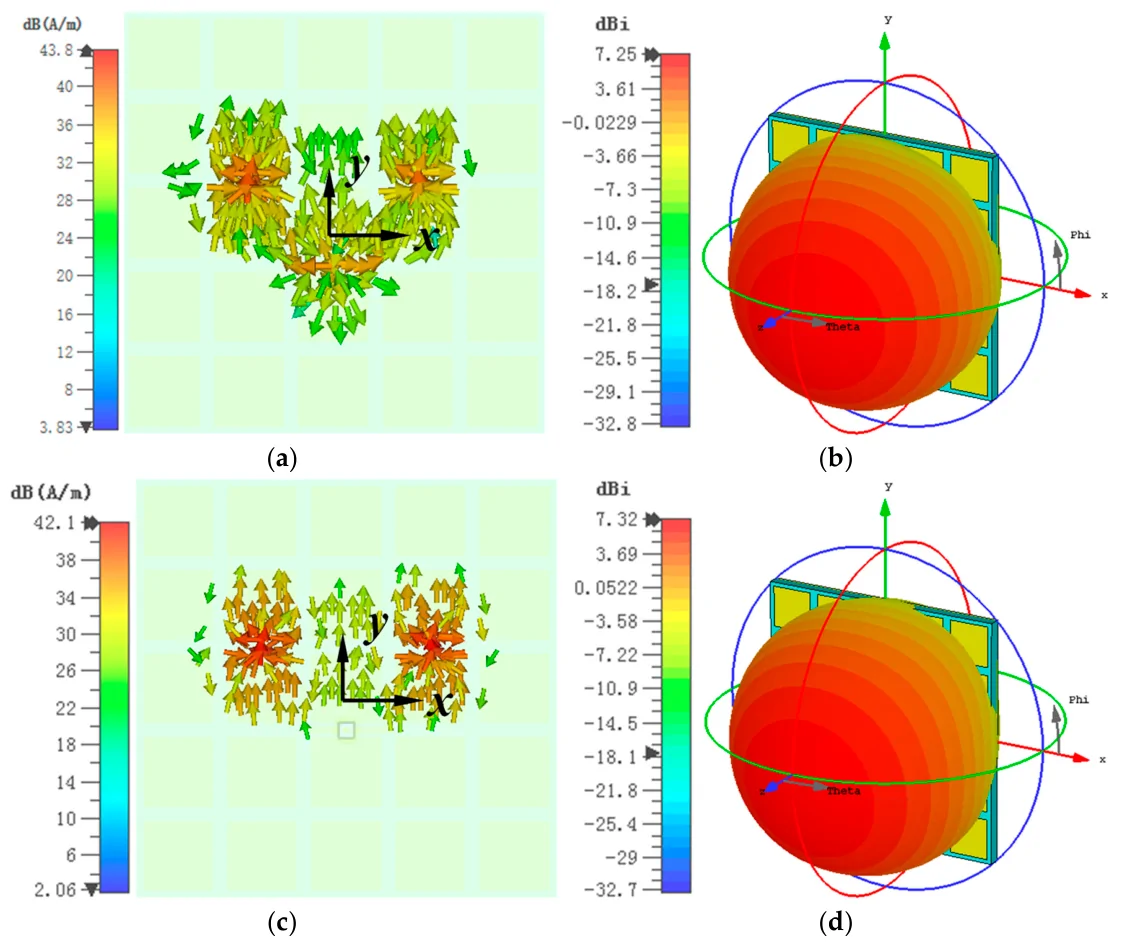
Radiation current distributions and radiation patterns: (**a**) radiation current at 3.0 GHz; (**b**) radiation pattern at 3.0 GHz; (**c**) radiation current at 3.08 GHz; (**d**) radiation pattern at 3.08 GHz.

Within the CMT framework, the excitation capability of a feeding structure for a given mode mainly depends on the spatial overlap and directional matching between the feed current and the target-mode current. Since the modal currents shown in Figure 5a,i are concentrated in the central region of the structure and are predominantly directed along the *y*-axis, an L-shaped feeding structure is adopted to excite these modes. The resulting configuration corresponds to antenna element 1, as shown in Figure 1c. The horizontal and vertical arms of the L-shaped feed can be adjusted to control the excitation position, equivalent current direction, and excitation strength, thereby enhancing the selective excitation of the target modes. With W fixed at 1.0 mm, Figure 6 presents the reflection coefficients for different values of V. When V = 1.0 mm, corresponding to the fully symmetric configuration with V = W, antenna element 1 exhibits single-band operation. As V decreases to 0.9 mm and 0.8 mm, two adjacent resonances emerge. As shown in Figure 7, when V = 0.8 mm, the two resonant frequencies occur at 3.0 GHz and 3.08 GHz. At both resonant frequencies, the radiation currents are predominantly directed along the *y*-axis and concentrated in the central patch region, while the main beams remain oriented in the broadside direction.

### 2.2. Design of Antenna Scattering Characteristics

Numerical evaluation of scattering performance consumes far more computational resources than assessing radiation performance, and conventional low-scattering antenna design typically demands iterative optimization rounds. As a result, such traditional design workflows suffer from low efficiency. To tackle this drawback, CMT is adopted to extract dominant scattering modes and their current-concentration zones prior to structural modification. This strategy lessens reliance on blind global optimization and boosts the physical interpretability and efficiency of the low-scattering design.

For this purpose, CMT was used to analyze the scattering modes of antenna element 1 under plane-wave illumination. For a finite-sized metasurface, an incident plane wave excites induced currents on the surfaces of the metal patches. According to CMT, the scattering-induced current can be expressed as a linear superposition of multiple characteristic modal currents:

(1) $$J^{S}=\sum_{n}^{N}\alpha_{n}^{S}J_{n}$$

where $J^{S}$ is the total scattering-induced current under plane-wave illumination, $J_{n}$ is the current of the n-th characteristic mode, $\alpha_{n}^{S}$ is the corresponding MWC, and N is the number of modes participating in the expansion. For plane-wave incidence, the MWC can be expressed as:

(2) $$\alpha_{n}^{S}=\frac{V_{n}^{i}}{1+j\lambda n}$$

where λn is the eigenvalue of the nth mode, and $V_{n}^{i}$ is the excitation intensity of the incident field for this mode. When λn approaches 0, the mode is in the resonant state, its modal significance is high, and it contributes substantially to the total scattering field. Therefore, the key to reducing RCS is to decrease the MWCs of the dominant scattering modes in the target frequency band.

**Figure 8:**
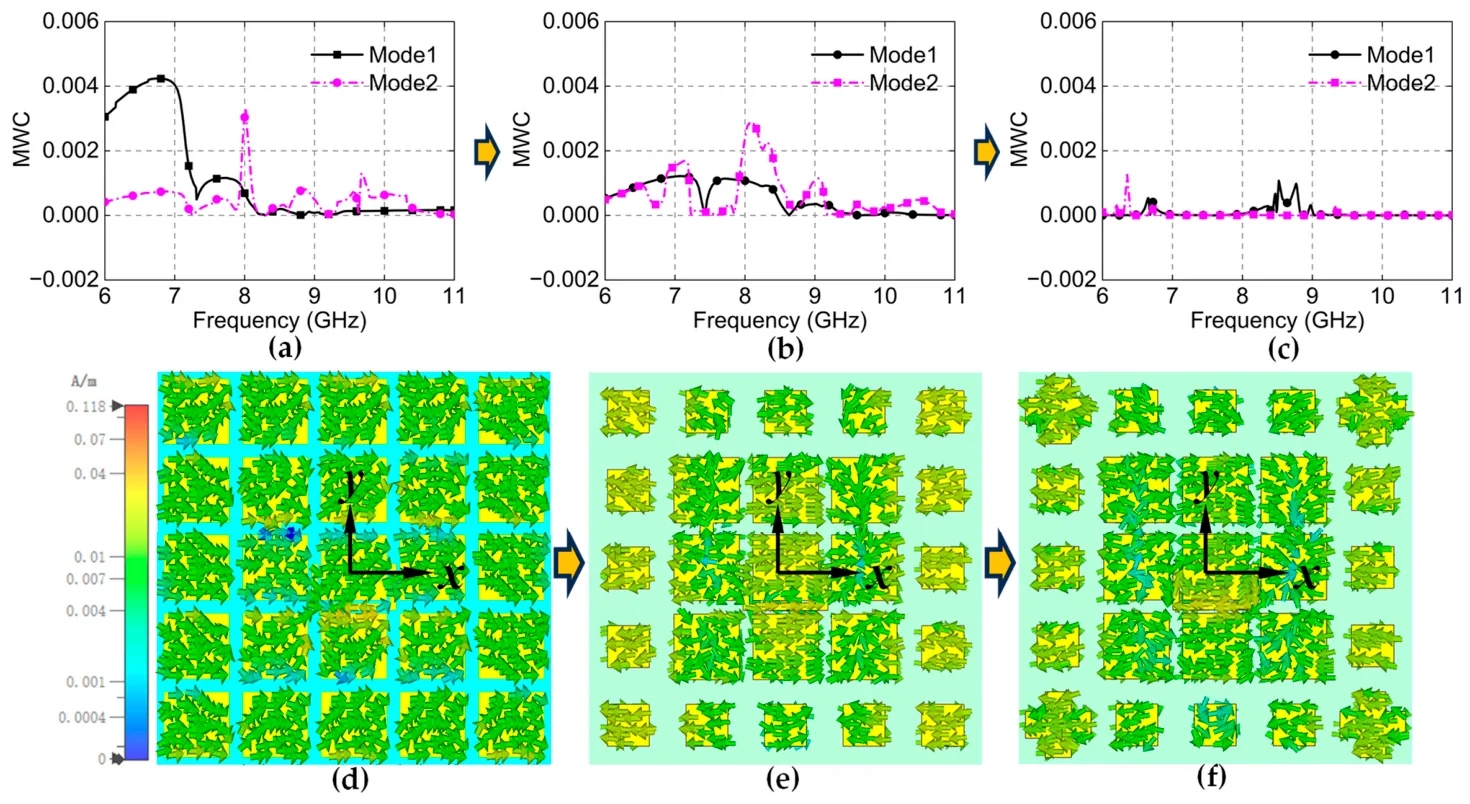

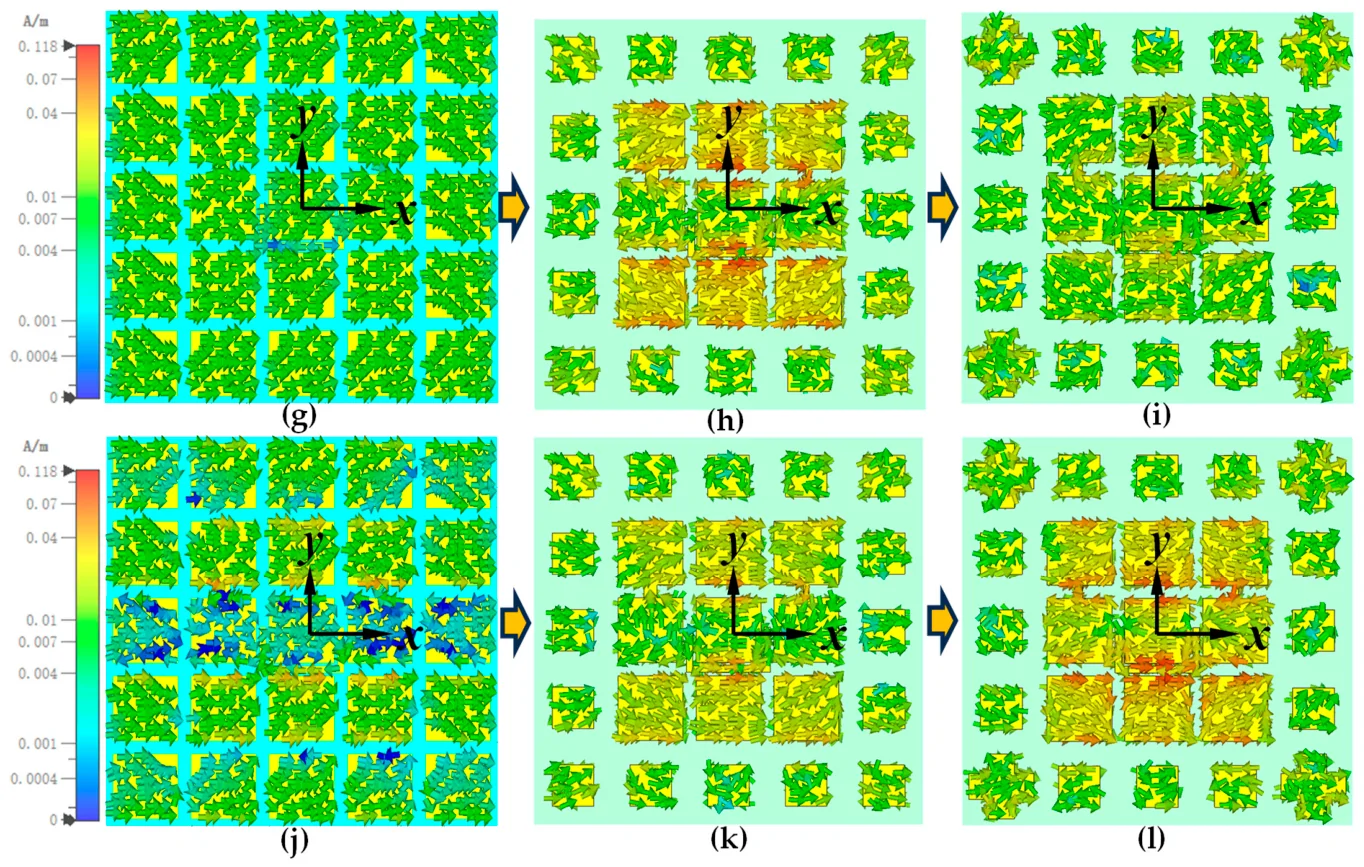
MWCs and scattering-mode current distributions of the three antenna elements under x-polarized plane-wave incidence: (**a**–**c**) MWCs of antenna elements 1–3, respectively; and (**d**–**f**), (**g**–**i**), and (**j**–**l**) scattering current distributions of antenna elements 1–3 at 6.8, 8.0, and 9.7 GHz, respectively.

Figure 8a presents the MWCs of antenna element 1 under x-polarized plane-wave illumination. Multiple prominent MWC peaks are observed within the 6.0–11.0 GHz band, primarily around 6.8 GHz, 8.0 GHz, and 9.7 GHz. The scattering modes associated with these MWC peaks make significant contributions to backscattering under plane-wave excitation. Accordingly, the low-scattering design focuses on structural modification at these frequencies and the corresponding current-concentration regions. To determine the regions to be modified, the scattering-mode current distributions at these target frequencies are further examined, as shown in Figure 8d,g,j. At the three target frequencies, the scattering-mode currents exhibit pronounced codirectional distributions, leading to coherent superposition in the backscattering direction. This observation indicates that antenna element 1 is prone to strong coherent backscattering at these frequencies. Accordingly, the corresponding scattering modes are selected as the primary suppression targets for the subsequent low-scattering design. The preceding radiation analysis shows that the radiation currents within the operating bands are mainly concentrated in the black-outlined region in Figure 1c. Therefore, the scattering-oriented structural modifications are designed to avoid the central radiation-current region as much as possible. To this end, all outer patches are uniformly reduced in size. The resulting increase in the gaps between the outer patches disrupts the continuity of induced currents in the outer-edge regions, while the geometry of the central patches remains unchanged. The resulting modified configuration corresponds to antenna element 2, as shown in Figure 1d.

After the first structural modification, the MWCs and scattering-mode current distributions of antenna element 2 are shown in Figure 8b,e,h,k. At 6.8 GHz, the MWC of antenna element 2 decreases compared with that of antenna element 1. However, relatively high MWC values remain at 8.0 GHz and 9.7 GHz. Further examination of the scattering-mode current distributions shows that the continuous currents on the outer-edge patches are weakened, whereas strong scattering-mode currents remain on the four corner patches, with their dominant directions approximately aligned with those on the central-region patches. To further suppress these scattering contributions, the four corner patches are reconfigured, resulting in antenna element 3, as shown in Figure 1e. In this design step, the size of the outer patches is fixed at 6.4 mm, while the four corner patches are reshaped into cross-shaped elements. These cross-shaped patches disrupt the continuous current paths supported by the original square corner patches and reduce the metallic filling area in the corner regions. Compared with the original square patches, the cross-shaped elements modify the induced-current paths within the four corner regions. Consequently, the corner currents no longer maintain continuous propagation along the positive *x*-axis, thereby weakening the codirectional superposition of the scattering fields from the corner and central regions. As shown in Figure 8c, the MWC values of antenna element 3 are significantly reduced at the target high-scattering frequencies. At 6.8 GHz, the scattering-mode current distribution shown in Figure 8f remains similar to that obtained after the first modification step. Oppositely directed scattering-mode currents are still distributed over the central region and portions of the outer regions. Accordingly, the RCS-reduction performance at this frequency remains comparable to that achieved after the first modification. In contrast, the additional suppression introduced by the four cross-shaped corner patches becomes more pronounced at 8.0 GHz and 9.7 GHz. As shown in Figure 8i,l, the corner-patch modification changes the directions of the scattering-mode currents, which are no longer aligned with those on the central-region patches. The corner currents that originally flowed predominantly along the *x*-axis are redistributed among different metallic branches of the cross-shaped patches, resulting in bent and rearranged local current paths. This current redistribution weakens the coherent superposition between the scattering fields generated by the corner and central regions, thereby reducing the total backscattered energy.

**Figure 9:**
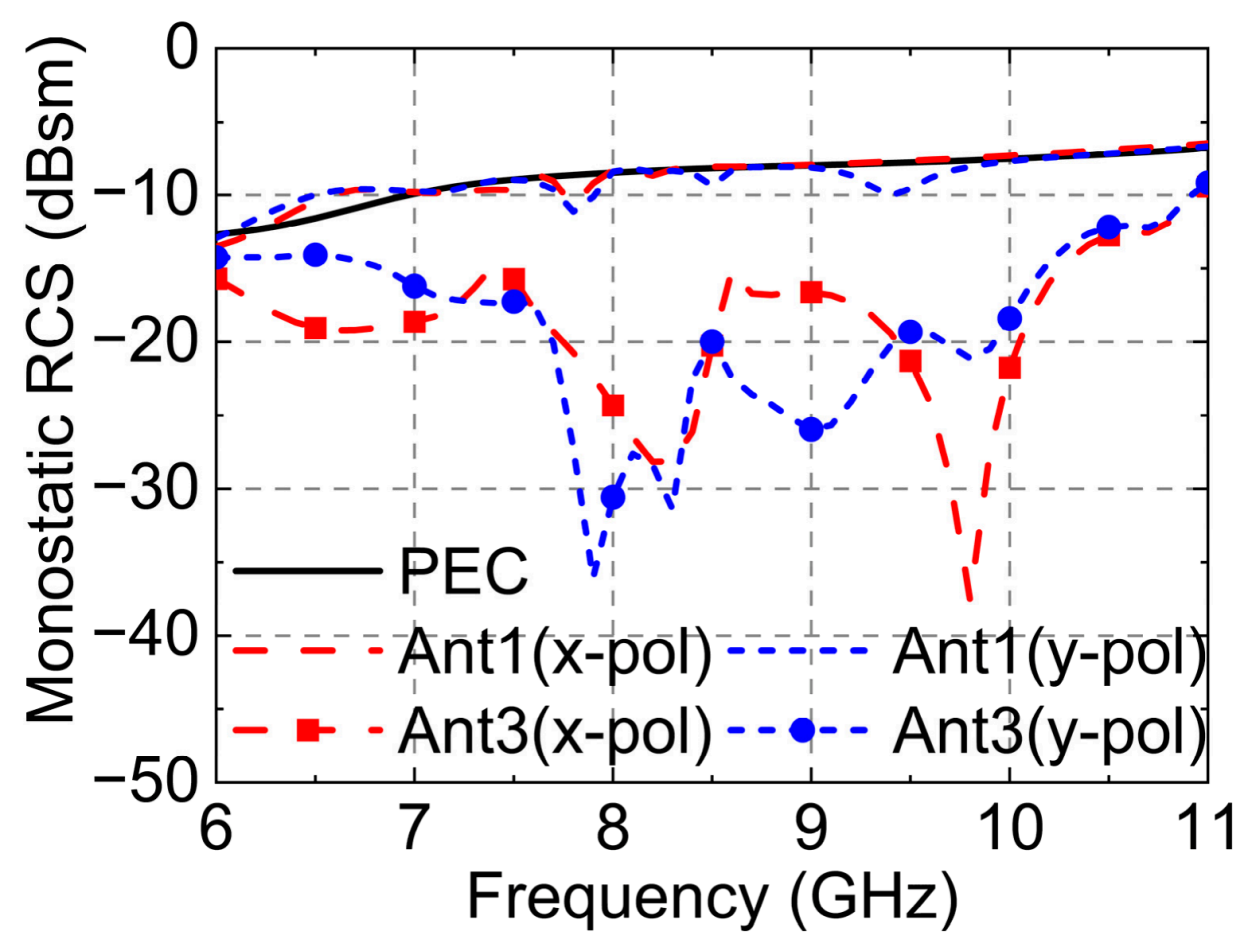
Monostatic RCS comparison of antenna elements 1 and 3 relative to a metal plate of the same dimensions.

**Figure 10:**
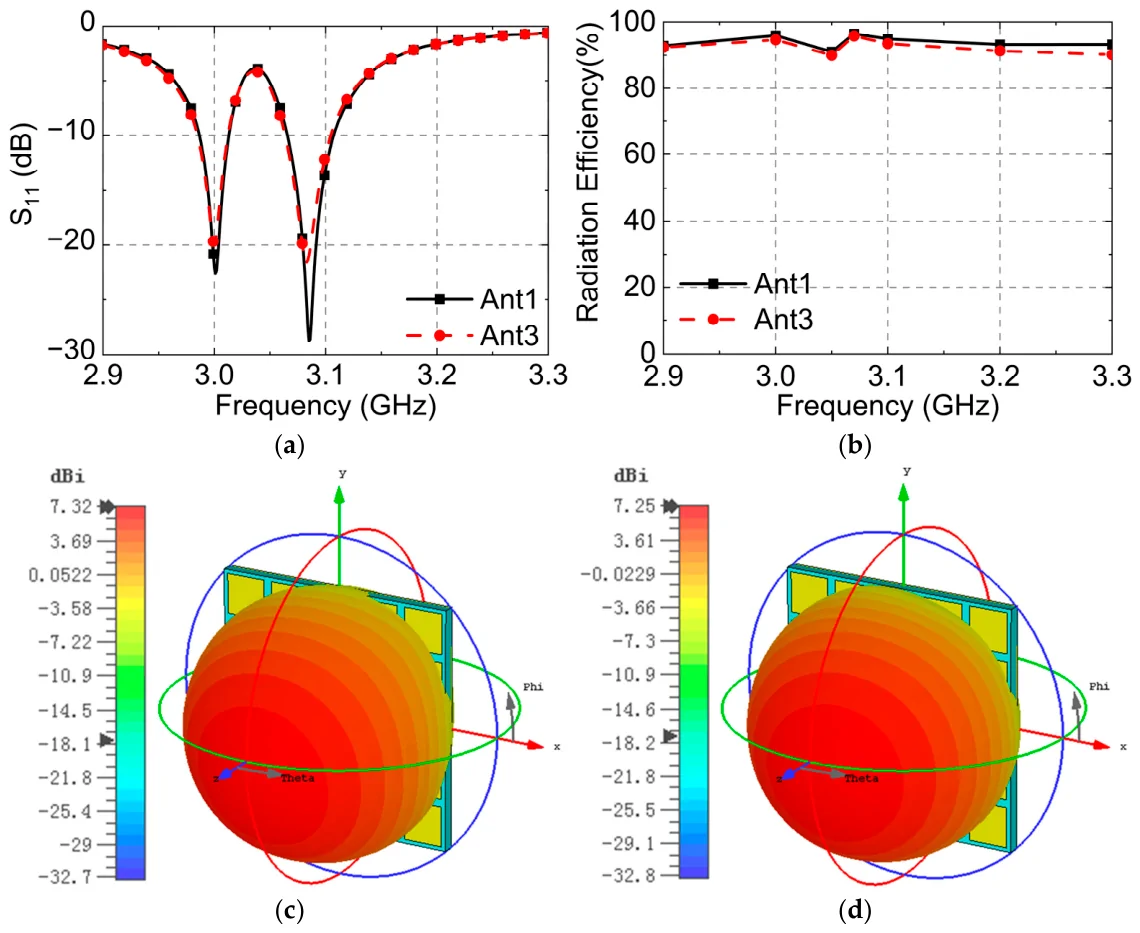
Radiation performance comparison of antenna elements 1 and 3: (**a**) reflection coefficients (S_11_); (**b**) radiation efficiencies; (**c**) radiation pattern of antenna element 3 at 3.0 GHz; and (**d**) radiation pattern of antenna element 3 at 3.08 GHz.

Figure 9 presents the monostatic RCS comparison of antenna elements 1 and 3 relative to a metal plate of the same dimensions. After antenna element 1 is modified based on the CMT-derived scattering-mode characteristics, antenna element 3 achieves broadband RCS reduction over the 6.5–10.5 GHz band. Furthermore, antenna element 3 retains an approximately symmetric configuration, and RCS reduction is observed under both x- and y-polarized plane-wave incidence. Meanwhile, the reflection-coefficient responses and radiation efficiencies of antenna element 3 remain nearly unchanged compared with those of antenna element 1, as shown in Figure 10a,b. The main beams of antenna element 3 remain oriented in the broadside direction at 3.0 GHz and 3.08 GHz, as shown in Figure 10c,d. These results indicate that CMT-guided scattering-mode identification and local structural modification can effectively suppress dominant scattering contributions while largely preserving the radiation performance within the operating bands.

### 2.3. Radiation and Scattering Performance of the Array Antenna

**Figure 11:**
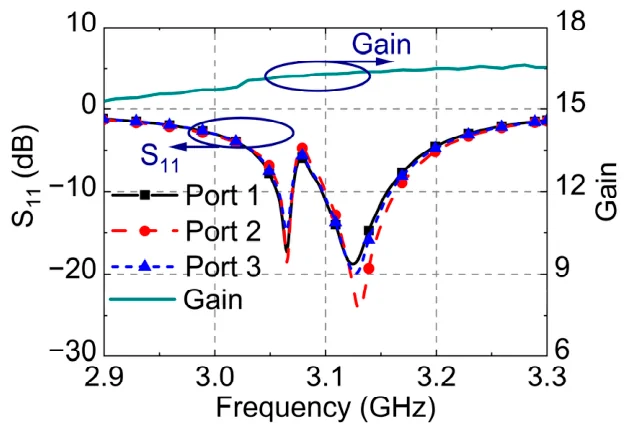
Gain and element reflection coefficients of the array antenna.

**Figure 12:**
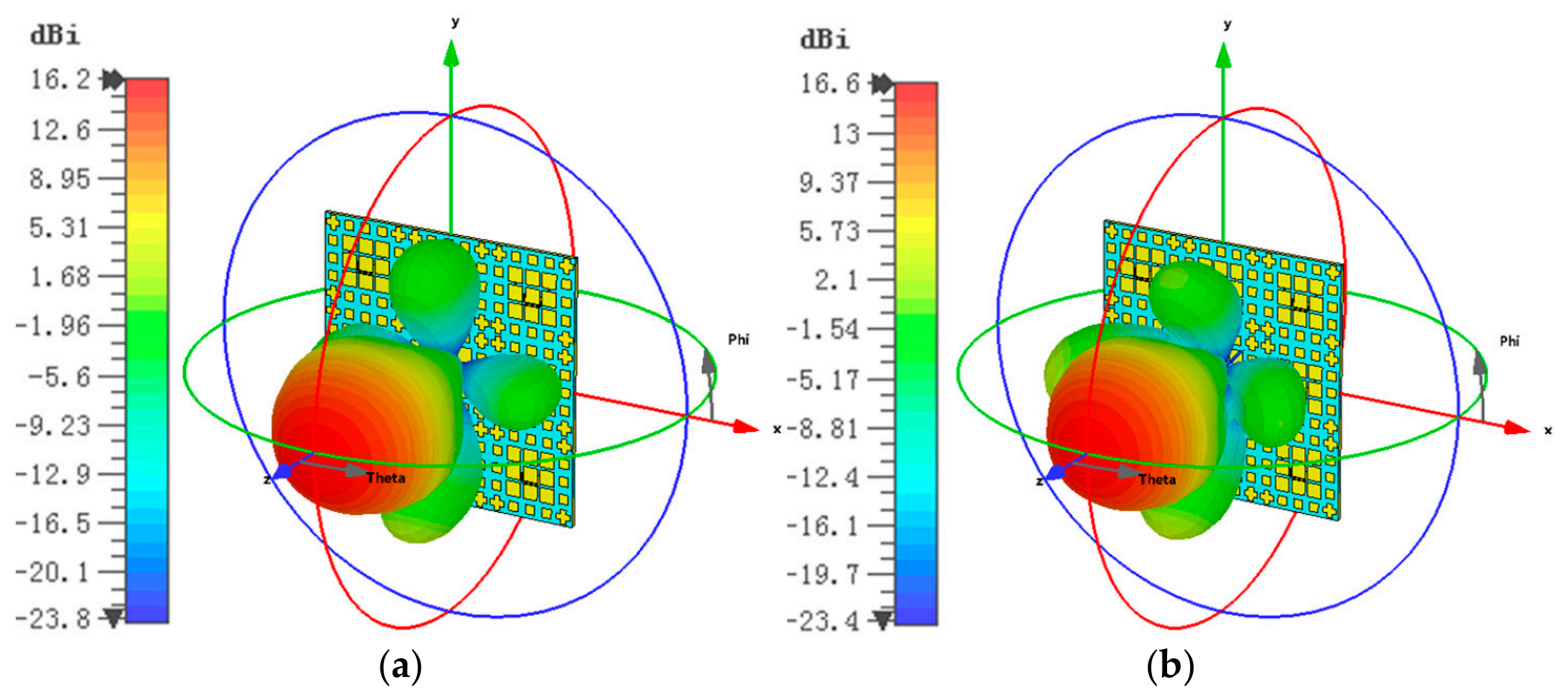
Far-field radiation patterns of the array antenna: (**a**) 3.07 GHz; (**b**) 3.13 GHz.

To further validate the effectiveness of the element design, a 3 × 3 array antenna is constructed using antenna element 3, as shown in Figure 1f. Figure 11 presents the element reflection coefficients and array gain. After array formation, the antenna elements maintain good impedance matching within the target operating bands, with −10 dB impedance bandwidths of 3.06–3.08 GHz and 3.11–3.15 GHz. Meanwhile, the array antenna achieves a peak gain exceeding 16.0 dBi in both operating bands. Figure 12 presents the far-field radiation patterns of the array antenna at 3.07 GHz and 3.13 GHz. The main beams remain oriented in the broadside direction at both frequencies, with peak gains of 16.2 dBi and 16.6 dBi, respectively. These results indicate that the antenna retains its broadside radiation capability after array formation.

**Figure 13:**
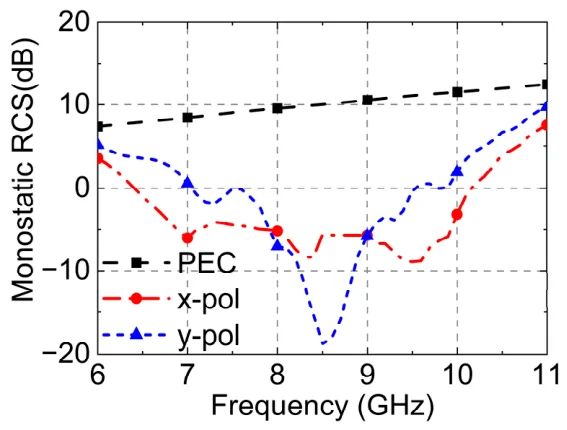
Monostatic RCS comparison of the array antenna relative to a metal plate of the same dimensions.

Figure 13 presents the monostatic RCS comparison between the array antenna and a metal plate of the same dimensions. Under x- and y-polarized plane-wave illumination, the backscattering of the array antenna is substantially reduced over a relatively broad frequency band. In summary, the proposed low-scattering array antenna achieves effective RCS reduction over 6.5–10.5 GHz while preserving dual-band impedance matching, broadside radiation, and high gain. These results further validate the effectiveness of the proposed CMT-guided structural modification method. The method primarily involves local modification of the outer-edge and corner regions to achieve broadband RCS reduction while preserving the established dual-band radiation characteristics, resulting in a structurally simple configuration. Furthermore, CMT provides a unified physical framework for radiation-mode selection, resonant-frequency tuning, scattering-mode identification, and RCS reduction. Accordingly, the proposed procedure provides a basis for extending the method to other dual-band low-scattering antenna configurations.

## 3. Measured Results

**Figure 14:**
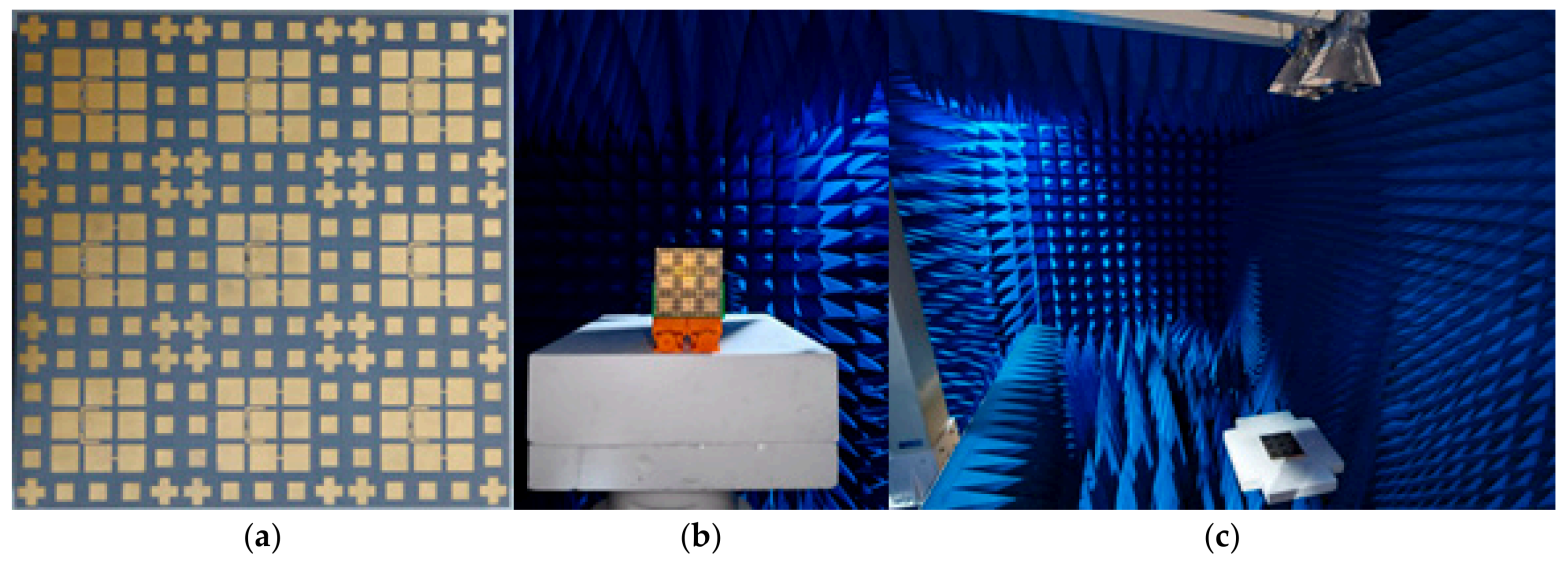
Fabricated array antenna and microwave anechoic-chamber measurement setups: (**a**) fabricated antenna prototype; (**b**) radiation-performance measurement setup; and (**c**) scattering-performance measurement setup.

**Figure 15:**
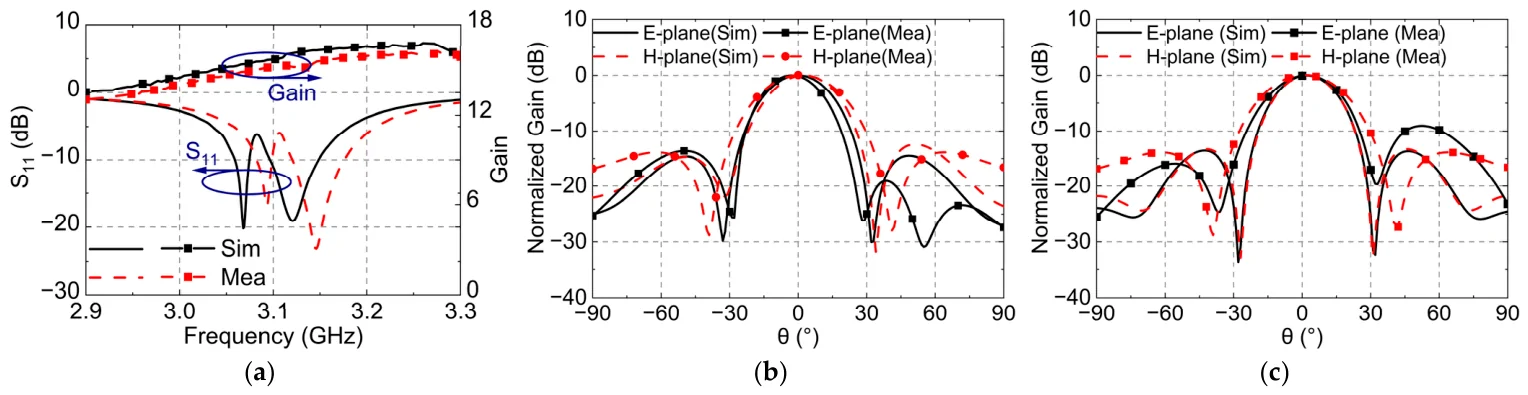
Simulated and measured radiation performance of the array antenna: (**a**) input reflection coefficients and gains; (**b**) normalized radiation pattern at 3.09 GHz; and (**c**) normalized radiation pattern at 3.14 GHz.

Finally, the array antenna shown in Figure 1f was fabricated, and its radiation and scattering performance was measured in a microwave anechoic chamber. A photograph of the fabricated prototype is shown in Figure 14a. The overall dimensions of the antenna are 180.0 mm × 180.0 mm × 3.0 mm. Figure 14b shows the radiation-performance measurement setup, while Figure 14c shows the scattering-performance measurement setup. Figure 15a compares the simulated and measured reflection coefficients and gains of the array antenna. The simulated resonant frequencies are 3.06 GHz and 3.11 GHz, while the measured resonant frequencies are 3.09 GHz and 3.14 GHz, respectively. Although upward frequency shifts of approximately 30 MHz are observed for both resonances, the measured results still exhibit the expected dual-resonance characteristics. These frequency shifts can be attributed to deviations of the actual substrate parameters from their nominal values, substrate-thickness tolerances, and other fabrication imperfections. At 3.09 GHz and 3.14 GHz, the measured gains reach 16.0 dBi and 16.4 dBi, respectively, showing reasonable agreement with the simulated results. Figure 15b,c show the normalized radiation patterns of the array antenna at 3.09 GHz and 3.14 GHz, respectively. The measured main beams at both frequencies remain oriented in the broadside direction. These results indicate that the low-scattering modifications do not significantly alter the broadside radiation characteristics associated with the target radiation modes. Accordingly, the array antenna maintains stable broadside radiation at the two operating frequencies. These experimental results validate the effectiveness of the CMT-guided radiation-mode selection and feeding design.

**Figure 16:**
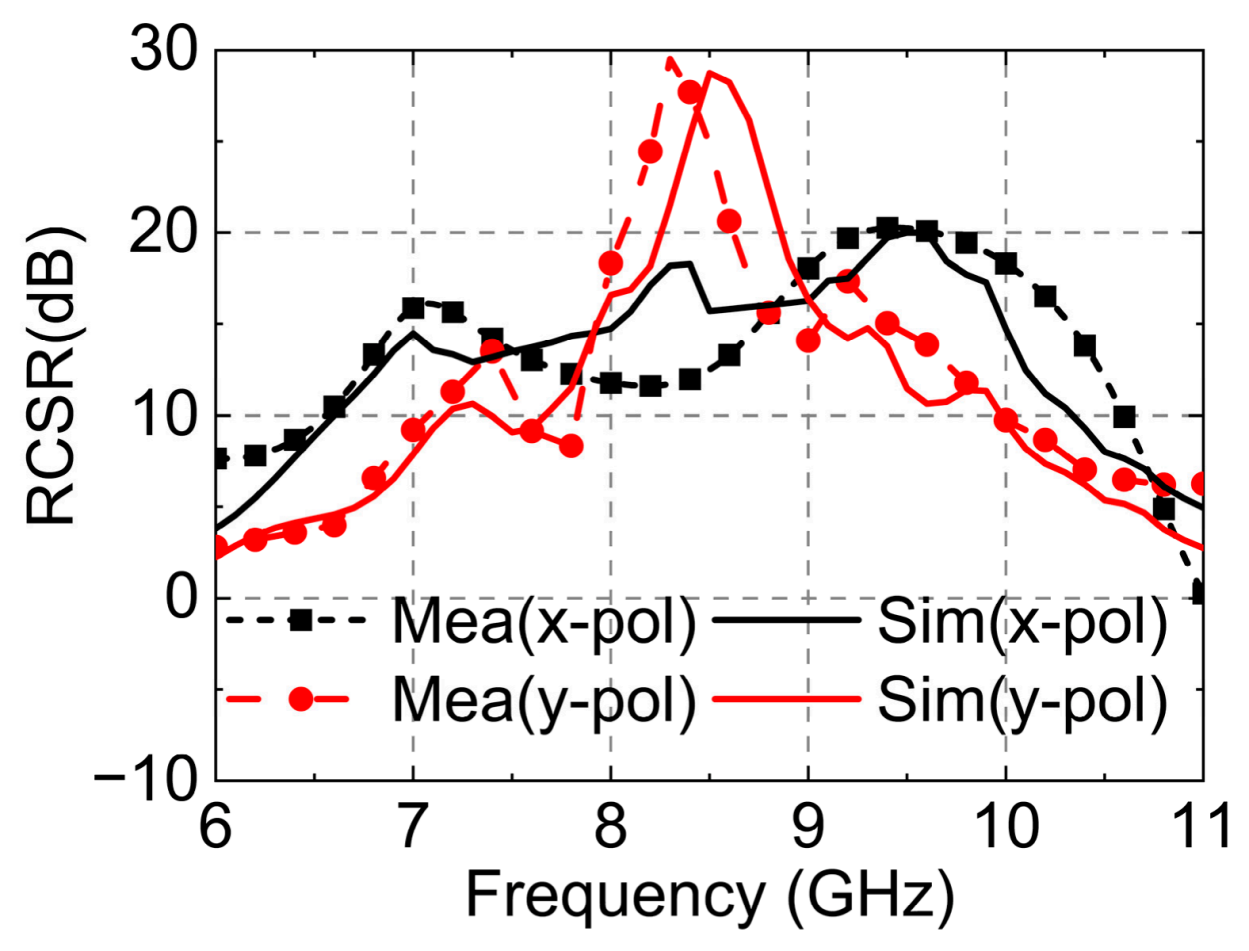
Monostatic RCS reduction relative to a metal plate of the same dimensions.

The monostatic RCS reduction in the array antenna relative to a metal plate of the same dimensions is shown in Figure 16. The measured results indicate that the designed array antenna achieves more than 10 dB RCS reduction over 6.5–10.5 GHz. Under x-polarized plane-wave incidence, the maximum measured RCS reduction reaches 20.5 dB, while under y-polarized plane-wave incidence, it reaches 29.5 dB. The measured results show reasonable overall agreement with the simulated trends, although noticeable discrepancies remain in the RCS responses. These discrepancies can be attributed to several factors, including fabrication tolerances of the prototype, additional scattering from connectors and solder joints, the finite measurement distance, misalignment between the antenna prototype and the reference metal plate, and background reflections within the anechoic chamber. Despite these discrepancies, the measured results indicate that the proposed array antenna achieves RCS reduction over a relatively broad frequency band while preserving dual-band broadside radiation.

In summary, the measured results validate the effectiveness of the proposed design method. The fabricated array antenna maintains stable broadside radiation at the two operating frequencies while achieving broadband RCS reduction.

Table 1 compares the key performance metrics of the low-scattering dual-band antenna proposed in this work with those of previously reported antennas. The comparison shows that the proposed antenna does not outperform existing designs in every performance metric. Its primary contribution lies in proposing a CMT-guided design approach for low-scattering dual-band antennas and establishing a radiation–scattering co-design procedure driven by characteristic-mode parameters. Using this methodology, the designed antenna integrates dual-band high-gain radiation and broadband RCS reduction within a low-profile configuration without introducing additional superstrates or air cavities. The effectiveness of the proposed design method is further validated through prototype fabrication and experimental measurements.

The main limitation of the proposed antenna is the relatively narrow impedance bandwidths of its two operating bands. This limitation is primarily attributed to the current coplanar feeding structure. Following the proposed design methodology, the impedance matching can be further improved and the operating bandwidths broadened by optimizing the feeding structure. One feasible approach is to position the feeding structure beneath the radiating layer and excite the radiating patches through electromagnetic coupling. Such a configuration may introduce additional tunable resonances and is therefore expected to further broaden the impedance bandwidths of the antenna.

In summary, the primary objective of this work is not to optimize each individual performance metric independently, but to establish a design method for the joint control of dual-band radiation and broadband RCS reduction. Within this framework, radiation and scattering modes can be selectively identified and regulated according to specific requirements for operating frequency, gain, bandwidth, profile height, and low-scattering performance. Accordingly, the proposed method provides a feasible approach for developing dual-band low-scattering antennas for a wide range of application scenarios.

## 4. Conclusions

This work proposes a CMT-guided co-design method for dual-band radiation and broadband RCS reduction in low-scattering antennas. The method exploits the differences in the spatial distributions of radiation and scattering characteristic modes. The central region of the metasurface is used to construct and excite dual-band radiation modes, while the outer-edge and corner regions are used for scattering-mode regulation. Based on this principle, a complete design procedure driven by characteristic-mode parameters is established. First, the target radiation modes are selected and regulated based on their modal significance, radiation-mode current distributions, and modal radiation patterns, and the corresponding feeding configuration is then determined. Next, the dominant scattering modes are identified using MWCs under plane-wave illumination. The corresponding scattering-mode current distributions are then used to determine the regions to be modified and the associated structural modification strategies. As a result, a low-scattering dual-band antenna design procedure with well-defined physical mechanisms and a coherent sequence of design steps is established for the joint control of radiation and scattering performance.

The metasurface antenna designed using the proposed method integrates dual-band radiation and broadband RCS reduction within a compact, low-profile configuration. The measured results show that the antenna operates over two frequency bands of 3.08–3.10 GHz and 3.12–3.18 GHz, with peak gains of 16.0 dBi and 16.4 dBi, respectively. Over the 6.5–10.5 GHz frequency range, the designed antenna achieves more than 10 dB monostatic RCS reduction under both x- and y-polarized plane-wave incidence. These experimental results validate the effectiveness of the proposed CMT-guided design method for low-scattering dual-band antennas.

Nevertheless, the two operating bands of the fabricated prototype remain relatively narrow. Future work will focus on further optimizing the feeding structure and extending the proposed method to antenna designs with broader impedance bandwidths and larger frequency ratios between the two operating bands.

## Figures and Tables

**Table 1:** Performance and design-feature comparison between the proposed antenna and recently reported antenna designs.

Ref.	Antenna Size (mm^3^)	Operating Band(s) (GHz)	Peak Gain (dBi)	RCS Reduction Band(s) (GHz)	Maximum Monostatic RCS Reduction (dB)	Structural Complexity *	Extent of CMT Use
[11]	160 × 160 × 28	5.10–5.40; 6.60–7.02	17.5; 16.9	9.40–13.70	21.0(x-pol.); 21.6(y-pol.)	High	None
[14]	108 × 108 × 34	4.15–4.80; 7.80–8.40	10.0; 15.9	3–4.8; 7.2–10.2; 10.8–16	15.1(x-pol.); 12.2(y-pol.)	High	None
[15]	240 × 240 × 36.6	0.85–0.93; 2.38–2.50	8.3; 10.9	0.745–1.02; 2.10–2.68;	29.5(x-pol.); 15.1(y-pol.)	High	None
[24]	160 × 160 × 7	4.35–4.45	13.5	3.25–6.60	25.9(x-pol.); 25.2(y-pol.)	High	Radiation & scattering
[25]	56 × 56 × 7	8.87–9.78	13.4	8.87–9.78	16(x-pol.)	High	Radiation & scattering
[26]	81 × 81 × 3.5	4.20–5.10; 5.60–6.50	9.5; 6.5	5.0–7.0	22.5(x-pol.); 20.0(y-pol.)	High	None
This work	180 × 180 × 3	3.08–3.10; 3.12–3.18	16.0; 16.4	6.5–10.5	20.5(x-pol.); 29.5(y-pol.)	Low	Integrated radiation–scattering co-design

* Low: single-layer integrated aperture without additional functional layers, air cavities, lumped elements, or active components; High: multilayer functional structures, F–P cavities, FSRs, lumped resistors, or active/bias components.

## Data Availability

The original contributions presented in this study are included in the article. Further inquiries can be directed to the corresponding authors.
